# Self-Disinfecting Paints with the Natural Antimicrobial Substances: Colophony and Curcumin

**DOI:** 10.3390/antibiotics10111351

**Published:** 2021-11-05

**Authors:** Micaela Machado Querido, Ivo Paulo, Sriram Hariharakrishnan, Daniel Rocha, Nuno Barbosa, Rui Galhano dos Santos, João Moura Bordado, João Paulo Teixeira, Cristiana Costa Pereira

**Affiliations:** 1 Environmental Health Department, National Institute of Health, 4000-055 Porto, Portugal; micaelaquerido@hotmail.com (M.M.Q.); cristiana.pereira@insa.min-saude.pt (C.C.P.); 2 EPIUnit, Institute of Public Health, University of Porto, 4050-600 Porto, Portugal; 3 Laboratory for Integrative and Translational Research in Population Health (ITR), 4050-600 Porto, Portugal; 4 Instituto Ciências Biomédicas Abel Salazar, University of Porto, 4050-313 Porto, Portugal; 5 CERENA—Centre for Natural Resources and the Environment, Instituto Superior Técnico, 1049-001 Lisboa, Portugal; ivo.p1691@gmail.com (I.P.); sriram.hariharakrishnan@tecnico.ulisboa.pt (S.H.); rui.galhano@ist.utl.pt (R.G.d.S.); jcbordado@ist.utl.pt (J.M.B.); 6 Barbot—Indústria de Tintas, S.A., 4410-295 Vila Nova de Gaia, Portugal; danielrocha@barbot.pt (D.R.); nunobarbosa@barbot.pt (N.B.)

**Keywords:** colophony, curcumin, paint, antimicrobial, self-disinfecting, cytotoxicity

## Abstract

The risk of infection arising from indirect sources—namely, contaminated surfaces—has been proved, particularly in healthcare facilities. In the attempt to minimize this problem, innumerable research projects involving the development of surfaces with self-disinfecting properties are being conducted. In this work, wall-paints with self-disinfecting properties were developed with the scope of being applied in environments prone to contamination, such as those at healthcare settings. Our approach was to develop new paint formulations containing two natural plant-based products with known antimicrobial activity—colophony (CLF) and curcumin (CUR). The natural substances were separately incorporated on a commercial paint and their antibacterial activity was evaluated with several bacterial species following ISO 22196. To assess the paints’ safety, cytotoxicity tests were performed on HaCaT and A549 cell lines, using tests on extracts and direct contact tests, as suggested by the standardized protocol ISO 10993. In general, both paints containing CLF and CUR were able to reduce the bacterial growth after 24 h, compared with the control, the commercial unmodified paint. Colophony was even able to reduce the number of culturable bacteria by over 2 log for *Staphylococcus aureus*, *Escherichia coli*, and *Bacillus cereus*. Regarding the cytotoxicity tests performed (WST-1, NRU, and LDH), both formulations revealed promising results regardless of the methodology used.

## 1. Introduction

Contamination of environmental surfaces is a serious public health problem with increasing interest over recent years, and with even more interest since the beginning of the COVID-19 pandemic [1,2]. Surface contamination with pathogens represents considerable risks for people’s health and is one of the major causes of infections spreading among individuals. This issue is even more worrying within healthcare settings, due to the great number of susceptible and frail people present in these spaces [3]. In the attempt to minimize surface contamination in those settings, several studies involving the development of surfaces with self-disinfecting properties have been conducted [4]. Surfaces have been modified to obtain antimicrobial properties in innumerable studies; however, dangerous chemicals are often applied, representing a potential risk for people contacting the surfaces directly or over longer periods of time. Moreover, increasing environmental awareness has led to the demand for less toxic and more ecological materials, including materials developed through science.

Consequently, there is a growing interest in natural products with antimicrobial properties that can be applied on surfaces and products. There are several natural and plant-based substances with known antimicrobial activity; colophony (CLF) and curcumin (CUR) are two of them.

CLF, also known as rosin, is a natural resin obtained from coniferous trees, such as pines, that can be easily modified to obtain innumerable products, such as curing agents, surfactants, adhesives, medicines, and biocides. From the industrial process, two main types of CLF are obtained: gum rosin, which is a residue that remains after distillation of tree resin, and tall oil rosin, which is a by-product of wood pulping in the kraft process [5].

CLF is considered safe, and it is applied in several cosmetic products, such as hair dyes, shaving creams, and other hygiene products, although some of its derivates may cause allergies or be skin sensitizers [6,7]. Furthermore, CLF and its derivatives are highly used as a coating agent in the pharmaceutical industry. Being a natural polymer, CLF has been used for drug delivery applications, either as microencapsulation material or as a binding agent in tablets [8,9]. Moreover, CLF derivatives have revealed good potential for application as chemotherapeutic agents since they showed cytotoxicity against cancer cells without increased cytotoxicity in normal cells [10].

Regarding the antimicrobial properties of CLF, some studies have already demonstrated that CLF and its derivatives have antimicrobial potential against different bacteria [11,12]. Niu et al. proved the antimicrobial performance of CLF incorporated on cellulose nanofibers against *Escherichia coli* and *Bacillus subtilis* [13]. Moustafa et al. also blended CLF with biopolymers (polylactic acid and poly(butylene adipate-co-terephthalate)) to obtain a food package material with antimicrobial properties [14].

CUR is the main component of the rhizome of the plant *Curcuma longa*, also known as turmeric. The medicinal properties of turmeric have been known for centuries, even though only more recently have the exact mechanisms and properties of this plant received more attention from the scientific community [15]. CUR has been broadly studied for its medicinal properties, and different studies demonstrated its anti-cancer, anti-infective, and antimicrobial properties, among others [16,17,18].

Over recent years, not only has CUR been evaluated in several clinical trials as a therapeutic agent but also different formulations of CUR have been studied and developed, such as capsules, tablets, nanoparticles, or powders, with a great number of these products being commercially available nowadays as supplements [19,20].

Regarding the antimicrobial properties of CUR, several studies have revealed the antiviral [21,22], antibacterial [23,24] and antifungal [25,26] activity of CUR.

The incorporation of CUR on several materials to obtain products with antimicrobial properties from a natural source has also been extremely studied more recently.

Qu et al. developed a micelle/hydrogel composite loaded with CUR to be applied as wound dressing. The final product presented antibacterial properties, as well as good biocompatibility and hemostatic properties, accelerating wound healing in in vivo studies [27]. Kulkarni et al. also developed a wound healing system containing CUR. In this case, thiocarbohydrazide gelatin nanofibers together with CUR were used to produce a nanofibrous material by electrospinning. The fibrous material containing CUR exhibited good antibacterial properties and was revealed to show good promise as a wound healing agent [28]. Gayani et al. developed a nanohybrid incorporating CUR and layered double hydroxide that revealed good antibacterial activity against *Pseudomonas aeruginosa*, *Staphylococcus aureus* and *Enterococcus faecalis*, showing even some antibiofilm activity [29].

With CLF and CUR being two natural substances with proven antimicrobial activity against different microorganisms, this study aimed at testing its application as a paint additive to create paints with self-disinfecting properties. The main goal of this work was to develop paints using natural products with antimicrobial activity—in this case, CLF and CUR—that could be applied in healthcare facilities. To do so, these substances were added, separately, to an acrylic commercial paint, and the antibacterial activity of the obtained paints was assessed following ISO 22196 and JIS Z2801 [30,31]. Since most studies involving surface modifications with antimicrobial substances do not present or even consider toxicity studies, we aimed to obtain an efficient but safe paint, assessing the toxicity of the paints containing CLF or CUR in HaCaT and A549 human cell lines to evaluate the products’ safety, following ISO 10993 with some adaptations [32]. The selected cell lines used in this work, HaCaT and A549, are skin and lung cell lines, respectively, representing the main routes of exposure to paints, either by skin contact or by inhalation [33,34].

## 2. Results and Discussion

### 2.1. Antibacterial Activity

The paint samples were considered to have antibacterial activity when presenting a value of R equal or superior to 2, according to JIS Z 2801 recommendations [31].

The paint with CLF presented antibacterial activity (R ≥2) against *S. aureus*, *E. coli*, and *B. cereus* (Table 1); however, it did not show antibacterial activity (R < 2) against *E. faecalis* and *K. variicola*. The paint containing CUR did not show antibacterial activity (R ≥2) against any of the bacterial species, although there was almost a two-log reduction for some species (*S. aureus, B. cereus*, and *E. faecalis*).

**Figure 1:**
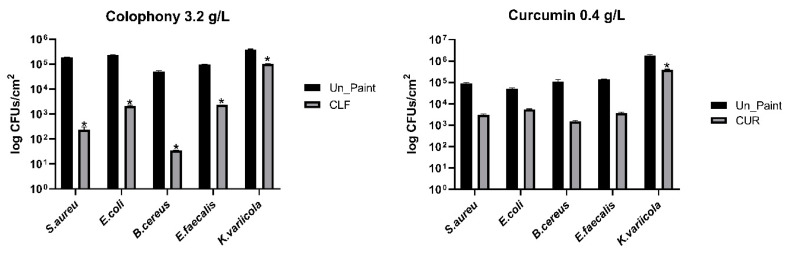
Average number of viable CFUs/cm^2^ recovered from each paint sample after 24 h of contact. The values are presented in logarithmic scale. The values are expressed as mean ± standard deviation. The statistical significance of samples (CLF/CUR) compared with Un_Paint is represented by *. (Two-way ANOVA; *p* < 0.05.)

Nonetheless, after 24 h both paints, containing CLF or CUR, reduced the number of viable bacteria, compared with the control, for all bacterial species (Figure 1).

Regarding surfaces containing the CLF, the mechanisms of action of CLF against bacteria were related to the interaction of the substance with the bacterial cell wall, although these mechanisms are not entirely known.

Despite some earlier studies asserting that CLF only presents antimicrobial activity against Gram-positive bacteria [35], the latest studies prove its antibacterial efficacy against Gram-positive and Gram-negative bacteria [36,37]. We conducted extensive research but no studies were found where the minimum inhibitory concentration (MIC) was assessed for CLF using any of the bacteria we used in this study. However, we found some studies involving CLF and its antimicrobial efficacy.

In a study carried out by Sipponen et al., colophony was able to inhibit the growth of several microorganisms, such as *S. aureus*, *B. subtilis*, *E. coli*, *P. aeruginosa*, and *Candida albicans* in a dose and time-dependent manner [36].

Kanerva et al. also developed polyethylene fibers incorporating colophony, which by showing inhibition zones on agar plates containing bacteria proved their good antimicrobial activity against *E. coli* and *S. aureus* [37].

De Castro et al. also successfully immobilized colophony on cellulose nanocrystals, improving their antimicrobial activity against *E. coli* and *B. subtilis* [38]. The methodology used to evaluate the antimicrobial activity was very similar to the methodology used in our work.

In relation to the CUR-containing surfaces, several previous studies proved the good antimicrobial activity of surfaces functionalized with CUR—namely, films and nanofibers. These surfaces had different purposes, such as food packaging or wound healing [28,39,40].

The CUR mode of antibacterial action is closely related to bacterial cell membrane interaction.

Mun et al. showed that the antibacterial effect of CUR on bacteria was related to the ability of increasing the cell wall permeability, which not only caused damage to the bacteria but also increased their sensitivity to other antibiotics, causing a synergistic effect [41]. Additionally, interacting with the bacterial cell wall, CUR can interact with bacterial enzymes and proteins. CUR has the capacity to bind to microtubulins blocking cell division and to disrupt RNA, inhibiting protein synthesis [42].

Gram-positive and Gram-negative bacteria have membranes with very different composition and structure, so the effect of CUR on each type is very different.

In this work, regarding the samples with CUR, the results show a higher sensitivity for Gram-positive bacteria, compared with Gram-negative (*B. cereus* > *E. faecalis* > *S. aureus* > *E. coli* > *K. variicola*), in agreement with the results found by Gayani et al. [29].

*B. cereus* was the bacterial specie that showed more sensitivity for CUR-containing samples, and according to previous studies, *B. cereus* indeed showed a high susceptibility when treated with CUR [43,44].

Wang et al. found that the CUR MIC value for *B. cereus* was 0.125 mg/mL [43] and that this value was the same found by Silva et al. in a similar study. *B. cereus* presented a low MIC value for CUR compared with other bacteria used in our tests, such as *S. aureus* 0.25–0.5 mg/mL or *E.coli* 0.25–0.5 mg/mL [44].

In the tests with CUR samples, *E. faecalis* showed similar values of susceptibility, compared with the other Gram-positive bacteria, *B. cereus* and *S. aureus*. According to the literature, *E. faecalis* presents a MIC for CUR of 0.5–0.625 mg/mL; however, for some strains of *E. faecalis*, this value can be lower (0.0625 mg/mL) [45,46].

Regarding the Gram-negative bacteria, *E. coli* and *K. variicola*, our R values were lower compared with the Gram-positive. As said before, the *E. coli* MIC values for CUR found in the literature fluctuated between 0.25 and 0.5 mg/mL; however, in a very recent work from Adamczak et al., testing the same *E. coli* strain used in our tests *E. coli* (ATCC 25922) found a much more elevated value of MIC (2 mg/mL). In this same study, median values of MIC of 2 mg/mL (the same as for *E. coli*) were found for *Klebsiella oxytoca* and for *Klebsiella pneumoniae* [45].

Comparing our results with the MIC values found in the literature and with the concentration of CUR in our samples, it is possible to understand that perhaps the concentration of CUR used (0.4 mg/mL) was not enough to obtain a greater antibacterial effect—namely, against *E. coli* and *K. variicola*, which present much higher MIC values according to the literature.

### 2.2. Test on Extracts

Following ISO 10993-5, during extract preparation, both serum-free and serum-containing medium can be used, although serum-containing medium is preferable since medium with serum can extract both polar and non-polar substances [32]. Furthermore, if a NRU assay is performed after exposure, serum must be used since its absence can affect cell growth; however, since serum proteins can mask toxicity, a serum concentration must be reduced to 5%. Therefore, during the performance of our assays, the serum concentration was reduced (from 10% to 5% FBS) in exposure medium.

**Figure 2:**
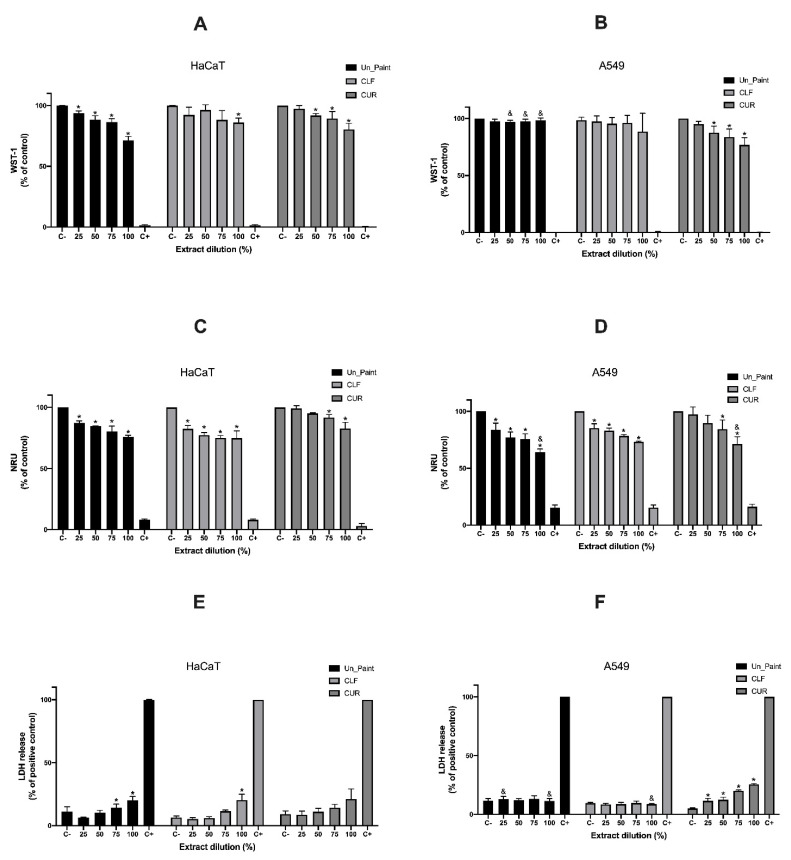
Results of WST-1 (**A**,**B**), NRU (**C**,**D**), and LDH (**E**,**F**) assays, with HaCaT and A549 cells, after 24 h incubation with CLF or CUR extracts at concentration of 25, 50, 75 and 100%. C-: negative control, C+: positive control. The values are expressed as mean ± standard deviation. The statistical significance of samples compared with C- is represented by *. (One-way ANOVA; *p* < 0.05). The statistical significance of A549 compared with HaCaT is represented by ^&^. (Two-way ANOVA; *p* < 0.05.)

Regarding the test on extracts performed using the HaCaT cell line (Figure 2A,C,E), the results of WST-1 revealed that Un_Paint presented a significant decrease in cell viability for all tested concentrations. Additionally, a significant decrease in cell viability was detected for CLF at 100% and for CUR at 50, 75, and 100%. However, the values of viability for the samples containing CLF or CUR were never below 80%, regardless of the tested concentration. According to ISO 10993-5, a reduction in cell viability by more than 30% is considered a cytotoxic effect [32].

Based on the results of NRU with HaCaT, the Un_Paint maintained a significant decrease in cell viability, as shown in WST-1 results. In this assay, CLF also showed a significant decrease in cell viability for all concentration, although the obtained values were over 75% of viability. CUR only presented significant reductions in viability for 75% and 100% concentration. For the NRU assay, CUR presented higher viability values (83–99%) compared with CLF (75–82%) or the Un_Paint (76–87%).

Concerning the HaCaT membrane integrity evaluated with LDH assay, the results showed that only control paint at 75% and 100% concentration and CLF at 100% concentration presented significant increases in LDH release. CUR did not present any significant increase, and the values for all tested samples were under 21%.

In general, the test on extracts performed on A549 cells (Figure 2B,D,F) revealed similar results to those obtained with HaCaT cells. The principal exceptions regarding the developed paints were CUR (100%) on NRU assay, which was significantly lower for A549 cells compared with HaCaT cells, and CLF (100%) for LDH assay on A549 cells, which was also significantly lower than on HaCaT cells.

The results of cellular viability on A549 obtained with the WST-1 assay revealed that control paint had no significant reductions, regardless of the concentration, as well as CLF substance. Nevertheless, CUR showed a significant decrease for 50, 75, and 100% concentrations.

The NRU assay on A549, the Un_Paint, and the paint containing CLF exhibited a significant reduction in cell viability for all concentrations. These results were in accordance with the ones obtained with NRU assay for HaCaT cells; however, they were slightly different from the ones obtained with WST-1 for A549 cells.

The results of WST-1 for A559 did not display the concentration gradient effect observed in the WST-1 assay with HaCaT cells or in NRU with both cell lines.

The samples containing CUR only presented a significant reduction for 75% and 100% concentration, showing the concentration gradient effect already observed in HaCaT cells or in WST-1 assay with A549 cells.

In the LDH assay with A549 cells, control paint and CLF once more displayed very similar results, with little variation with concentration increase; however, CUR-containing surfaces showed an increase in LDH leakage with increasing concentration and the values were significantly different from the negative control for all concentrations.

### 2.3. Test by Direct Contact

**Figure 3:**
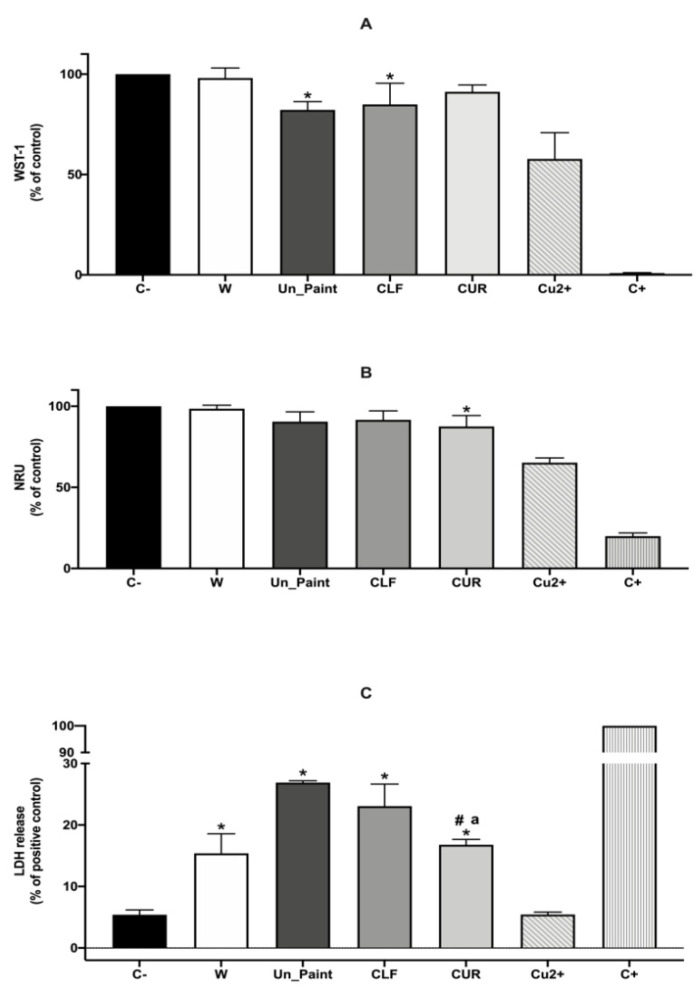
Results of WST-1 (**A**), NRU (**B**), and LDH (**C**) assays, with HaCaT cells, after 24 h of direct contact with CLF or CUR samples. C-: negative control, C+: positive control. The values are expressed as mean ± standard deviation. The statistical significance of samples compared with C- is represented by *, the statistical differences compared with Un_Paint are represented by ^#^, and the statistical differences compared with CLF are represented by ^a^. (One-way ANOVA; *p* < 0.05.)

Regarding the direct contact test performed with HaCaT cells (Figure 3), the WST-1 results revealed a good cellular viability for both the Un_Paint (82%) and the paints containing substances CLF (85%) and CUR (91%). However, the Un_Paint and CLF-containing samples revealed a significant decrease in cellular viability, while CUR value of viability was higher and not significantly different from the negative control.

On the other hand, the NRU results, although very similar to the results obtained with WST-1, showed a different outcome. The CUR-containing surface had a small but significant decrease in cellular viability, while Un_Paint and CLF presented no significant reductions. Nevertheless, it is important to consider that in addition to these statistical changes between assays, the range of obtained values of cellular viability with WST-1 (82, 85, and 91%) and NRU (90, 92, and 88%) was very similar, suggesting that both assays evaluating cellular viability presented similar outcomes.

The LDH assay after direct contact with the paints showed that all surfaces significantly increased the LDH release compared with the negative control. However, the surfaces containing CLF or CUR had a smaller increase in LDH release compared with the Un_Paint. CUR presented the better result, with only 17% of LDH leakage, a value that was statistically different from the one obtained for Un_Paint (27%) and from the one obtained for CLF (23%). The positive control of the surfaces (Cu^2+)^ showed low LDH release, opposite to what was expected; however, after some searching in the literature, we believe this may be related to the interference of the Cu^2+^ ions with the LDH [47,48].

Although the direct contact test reflects a more realistic scenario of exposure, representing the close contact between the paints and the cells, the preparation of extracts from the paints allows us to determine the biological reactivity of possible chemical leachables. Additionally, by performing extracts at different concentrations, it is possible to evaluate whether there is a dose-dependent reaction to the presence of the leachates from the test material—in this case, the paints. Comparing the two types of tests performed on HaCaT cells, extracts and direct contact, the LDH results were concordant. The values of LDH release, after direct contact with the paints or after incubation with extracts at 100% concentration, were approximate for both CLF (23% vs. 20%) and CUR (17% vs. 21%).

The NRU assays comparison, presented values of 75% vs. 92% for CLF and 83% vs. 88% for CUR. CLF presented distinctly higher percentages of cell viability after incubation with extracts, comparing to direct contact, according to NRU assay. However, the comparison of WST-1 assays realized on the distinct type of tests performed on HaCaT cells revealed values of 86% vs. 85% for CLF and of 80% vs. 91% for CUR, representing the extracts test and the test by direct contact, respectively.

In general, both of our paints showed low cytotoxicity towards HaCaT and A549 cells regardless of the methodology used. Regarding the CUR-containing products, previous studies had already demonstrated the low toxicity of CUR, especially when immobilized on materials. Kulkarni et al. demonstrated the low toxicity of nanofibers containing CUR using NIH3T3 cell line (fibroblasts). This study also revealed that CUR-containing fibers exhibited cytotoxicity in a dose-dependent manner, similar to what we realized in our study [28]. Gayani also proved the low toxicity towards the lung cell line MRC- 5 of a nanocomposite incorporating CUR [29].

## 3. Materials and Methods

### 3.1. Chemicals

Tryptic soy agar (TSA), maximum recovery diluent (MRD), tryptone soya broth (TSB), and plate count agar (PCA) were purchased from VWR (Radnor, PA, USA).

Trypsin-ethylenediaminetetraacetic acid (Trypsin-EDTA) 0.25%/1 mM EDTA 4Na in HBSS, w/o:Ca and Mg, w:Phenol red and Dulbecco’s modified Eagle’s medium (DMEM) with 4.5 g/L glucose and 2 mM L-glutamine were purchased from PanBiotech (Aidenbach, Germany). Fetal bovine serum heat inactivated (FBS) was purchased from Biowest (Nuaille, France) and 1% antibiotic-antimycotic solution from Corning (Corning-New York, NY, USA) as well as phosphate-buffered saline (PBS) 10× Molecular Biology Grade. Sodium dodecyl sulfate (CAS No. 151-21-3) was purchased from Merck (Darmstadt, Germany). Triton X-100 (CAS No. 9002-93-1) and Neutral Red (CAS No. 553-24-2) were purchased from Sigma-Aldrich (St. Louis, MO, USA). Lactate dehydrogenase (LDH) cytotoxicity detection kit and water-soluble tetrazolium (WST-1) cell proliferation reagent (CAS No.150849-52-8) were bought from Roche (Basel, Switzerland). CLF was purchased from Respol (Leiria, Portugal).

### 3.2. Paint Preparation

The CUR was obtained by extraction of from turmeric according to the protocol by Sahne et al. [49]. Acetone was used as solvent in the ultrasonic bath, at 35 °C for 35 min. The content was then filtered, with a 2 µm filter, and the solvent was removed by evaporation using a rotational evaporator. The obtained sample of CUR was dried using a freeze drier for 72 h. As previously noted, CLF was purchased.

The paints were prepared according to the procedure disclosed by Silva et al. [50] and Querido et al. [51]. Briefly, after the derivatization, the substances CLF and CUR were added to the commercial acrylic paint matrix (Velvet by Tintas Barbot). The mixing was performed in a mechanical stirrer with shear force at 800 rpm speed, for 5 min at RT and humidity conditions (25 °C, 50% HR).

The CLF and CUR were mixed at 3.2 g/L and 0.4 g/L, respectively. During the optimization process of paint preparations, different criteria were taken into account—namely, the paints’ color, viscosity, and opacity. Due to the low concentrations of the added antimicrobial substances, the color of the paints was only slightly affected. After incorporation of CLF, the paint color remained similar to the original. On the other hand, after incorporation of CUR, a slightly yellowish coloration was detected on the paint when in liquid state. However, after application on a clear-coated opacity chart (2A-H from Leneta) and drying, no differences were found in the color, compared with the original paint (data not shown). The Un_Paint presented values of whiteness of 78.58%, the CLF of 79.24%, and the CUR of 78.35%. The physical properties of the paints only suffered minor modifications after incorporation of the antimicrobial substances. The viscosity of the final formulations, containing CLF or CUR was slightly higher (376 cP and 376 cP, respectively), compared with the original paint (312 cP). The values of opacity were also similar for the three formulations, with the Un_Paint presenting a value of 99.28%, CLF a value of 99.29%, and CUR a value of 99.27%.

The equipment used for measuring the viscosity was Stormer Viscometer Myr VK2000 by Viscotech Hispania S.L. (Tarragona, Spain), and opacity and whiteness were analyzed using a DC400 spectrophotometer by Datacolor (Lawrenceville, NJ, USA). For utilization of our assays, the final formulations were then applied on polymeric coupons (50 mm × 50 mm or 10 mm × 10 mm), forming a layer of 200 µm of thickness. The drying time of the paint after application was 24 h.

### 3.3. Bacteria and Growth Conditions

For the antimicrobial testing of paints, two Gram-negative and three Gram-positive bacteria were selected: the Gram-negative *E. coli* (ATCC 25922) and *Klebsiella variicola* (ATCC 31488) and the Gram-positive *S. aureus* (ATCC 25923), *Bacillus cereus* (environmentally isolated from contaminated food in our laboratory), and *E. faecalis* (NCTC 775).

In addition to the bacterial species suggested by the ISO 222196 and JIS Z2801 standards (*S. aureus* and *E. coli*), we decided to test three more species frequently associated with hospital-acquired infections or environmental contamination (*B. cereus*, *E. faecalis*, and *K. variicola*).

Bacteria were grown on TSA plates overnight at 37 °C. Bacterial inoculum was prepared diluting some colonies in 9 mL of MRD to a cell density of 6 × 10^5^ colony forming units per milliliter (CFUs/mL), following MacFarland standard.

### 3.4. Antibacterial Activity

The antibacterial activity of the paints was assessed following ISO 22196 and JIS Z2801, with minor modifications [30,31].

The paint samples of (50 mm × 50 mm) unmodified commercial paint (Un_Paint) or containing CUR/CLF were sterilized using UV light for 15 min on each side. Afterwards, each paint sample was placed on a sterile Petri dish and inoculated with 400 μL of bacterial inoculum. The samples were covered with previously sterilized parafilm (40 mm × 40 mm) and incubated for 24 h at 37 °C with high humidity levels. As time zero control, a set of paint samples was treated right after inoculation with bacterial inoculum, without incubation.

After 24 h of incubation, 10 mL of a TSB neutralizing solution was added to each Petri dish, the parafilm was removed, and the Petri dish was gently stirred. Several dilutions from 10^−1^ to 10^−5^ were made using MRD and placed on sterile Petri dishes (n = 2 replicates were performed). Following, 15 mL of previously melted PCA was added to each Petri dish. After drying, the plates were incubated for 48 h at 37 °C with high humidity levels. Then, the number of CFUs on each plate was counted and the number of viable bacteria per cm^2^ per sample was calculated.

According to ISO 22196 and JIS Z 2801 [30,31], the value of antibacterial activity (R) was obtained following the equation:

(1) $$R=\left(U_{t}-U_{0}\right)-\left(A_{t}-U_{0}\right)=U_{t}-A_{t}$$

where R is the antibacterial activity; U_0_ is the average of the common logarithm of the number of viable bacteria, in CFUs/cm^2^, recovered from the control paint samples immediately after inoculation (T0); U_t_ is the average of the common logarithm of the number of viable bacteria, in CFUs/cm^2^, recovered from the control paint samples after 24 h (T24); A_t_ is the average of the common logarithm of the number of viable bacteria, in CFUs/cm^2^, recovered from the antimicrobial paint samples after 24 h (T24).

### 3.5. Cell Culture

HaCaT cells, a nontumorigenic immortalized human keratinocyte cell line, were kindly provided by the Aquatic Toxicology & Risk Assessment group from CESAM, University of Aveiro. The A549 cell line, a human alveolar epithelial cell line (ECACC 86012804; Human Caucasian lung carcinoma) was purchased from the European Collection of Authenticated Cell Cultures (ECACC, St. Louis, MO, USA).

Cells were cultured in DMEM supplemented with 10% (*v/v*) FBS and 1% antibiotic–antimycotic solution and grown at 37 °C, 5% CO_2_, in humidified atmosphere. The medium was changed every two days, and the culture was split using 0.25% trypsin-EDTA to detach cells when 80% confluency was reached.

### 3.6. Test on Extracts

Following ISO 10993-5 [32], test on extracts was performed with some modifications.

For extracts preparation, sterilized paint samples (10 mm × 10 mm) of unmodified paint (Un_Paint), CUR, and CLF were placed in 24-well plates, and 1 mL of medium with 5% FBS (exposure medium) was added. The plates were incubated for 24 h at 37 °C, 5% CO_2_, and after, different dilutions of the released extracts were performed. The original extracts (100%) were diluted to 75%, 50%, and 25%.

In parallel, cells were seeded at a concentration of 1.0 × 10^5^ cells/mL in 96-well plates (100 μL) and incubated for 24 h for adhesion at 37 °C, 5% CO_2_, in humidified atmosphere.

After the adhesion period, the medium was removed and replaced with the freshly prepared extracts (100%, 75%, 50%, and 25%), and cells were exposed for 24 h, at 37 °C, 5% CO_2_, in humidified atmosphere.

Next, cellular viability and membrane integrity assays were performed as described below.

The exposures were performed in triplicates on three independent experiments.

### 3.7. Test by Direct Contact

Following ISO 10993-5 [32], test by direct contact was performed with some modifications.

Cells were seeded at a concentration of 1.0 × 10^5^ cells/mL in 6-well plates (2 mL) and adhered for 24 h at 37 °C, 5% CO_2_, in humidified atmosphere.

After cell incubation, the culture medium was replaced with fresh exposure medium, and the paint samples (1 cm × 1 cm) of Un_Paint, CUR, and CLF were placed over the cell layer and gently pushed to come into direct contact with the cells. Samples of transparent polymeric film (W) and copper (Cu^2+^) were used as negative and positive control of the surface, respectively. The 6-well plates were then incubated for 24 h at 37 °C, 5% CO_2_, in humidified atmosphere.

Afterwards, the paint samples were gently removed from the surface of the cells, and cellular viability and membrane integrity assays were performed as described below.

The exposures were performed in triplicates on three independent experiments.

### 3.8. Cellular Viability (WST-1)

After exposure to extracts or after direct contact with the paint samples, cellular viability and metabolic activity were assessed following the WST-1 Cell Proliferation Reagent Kit (Roche, Basel, Switzerland). Untreated cells, only with exposure medium, were used as negative control, and cells treated with Triton X-100 solution (1%) were used as positive control.

After exposure, the supernatant was removed, and the cells were treated with 100 µL of WST-1 reagent (diluted 1:10 in serum-free medium) and incubated for 2 h at 37 °C, 5% CO_2_, protected from light. Optical density (OD) was measured at 450 nm (reference wavelength 630 nm) on a SpectraMax^®^ iD3 multi-mode microplate reader from Molecular Devices (San Jose, CA, USA). The results are expressed as percentage compared with the control,

(2) $$\left(\frac{{\mathrm{OD}}_{\mathrm{sample}}\;-\;{\mathrm{OD}}_{\mathrm{blank}}}{{\mathrm{OD}}_{\mathrm{control}}\;-\;{\mathrm{OD}}_{\mathrm{blank}}}\right)\times 100$$

### 3.9. Cellular Viability (NRU)

After exposure to extracts or after direct contact with the paint samples, cellular viability was assessed following the NRU assay, as suggested by ISO 10993-5 [32]. Untreated cells, only with exposure medium, were used as negative control, and cells treated with SLS (0.2 mg/mL) were used as positive control.

To perform this assay, the supernatant was discarded, cells were treated with NRU solution (diluted 1:10 in serum-free medium) and incubated for 3 h at 37 °C, 5% CO_2_, protected from light. After incubation, the solution was removed, cells were washed with PBS (100 µL/well), and then the desorption solution (49:50:1, water/absolute ethanol/acetic acid) was added (200 µL/well). The plate was shaken for 10 min, RT, and optical density (OD) was measured at 540 nm (reference wavelength 630 nm) on the SpectraMax^®^ iD3 multi-mode microplate reader from Molecular Devices (San Jose, CA, USA). The results are expressed as percentage compared with the control, following Equation (2).

### 3.10. Membrane Integrity (LDH)

After exposure, cellular membrane integrity was evaluated by LDH assay. Untreated cells, only with exposure medium, were used as negative control, and cells treated with Triton X-100 solution (1%) were used as positive control.

After exposure, the supernatant (100 µL/well) was placed on a 96-well plate and was centrifuged at 250× *g* for 10 min at room temperature (RT). Then the supernatant was moved to a new 96-well plate (50 µL/well) and incubated with LDH Cytotoxicity Detection Kit solution (in a ratio of 1:1) at RT, protected from light. After 20 min, the optical density (OD) was measured at 490 nm (reference wavelength 630 nm) on the SpectraMax^®^ iD3 multi-mode microplate reader from Molecular Devices (San Jose, CA, USA). The results are expressed as percentage compared with the positive control,

(3) $$\left(\frac{{\mathrm{OD}}_{\mathrm{sample}}\;-\;{\mathrm{OD}}_{\mathrm{blank}}}{{\mathrm{OD}}_{\mathrm{positive}\;\mathrm{control}\;}\;-\;{\mathrm{OD}}_{\mathrm{blank}}}\right)\times 100$$

### 3.11. Statistical Analysis

Data are reported as mean ± standard deviation (SD).

For antibacterial activity assessment, statistical significances between the number of CFUs/mL after 24 h of contact with the CLF/CUR and the number of CFUs/mL after 24 h of contact with the Un_Paint were analyzed, for the five bacterial species. The results were analyzed by two-way ANOVA followed by Sidak’s multiple comparison test, with the two factors being the type of paint and bacterial specie.

For test on extracts and test by direct contact, statistical significances of data against negative control (C-) were analyzed by one-way ANOVA followed by Dunnett post hoc test. Statistical differences between different paints were analyzed by one-way ANOVA followed by Tukey’s test.

Statistical differences for the same paint, on the same concentration but for different cell lines (comparing A549 and HaCaT), were analyzed by two-way ANOVA followed by Sidak’s multiple comparison test, with the two factors being the cell line and the concentration of the paint extract.

The differences were considered statistically significant at *p* < 0.05. The statistical analyses were performed using Graph Pad Prism 8.0 (GraphPad Software, San Diego, CA, USA).

## 4. Conclusions

The obtained results show that both developed paints, either containing CUR or CLF, presented antibacterial effects. The paint with CLF presented better results, while the paint with CUR presented more modest effects. Moreover, CLF was effective against Gram-positive and Gram-negative bacteria.

According to the requirements of JIS Z 2801, the paint containing CLF presented antibacterial activity (R > 2) against *S. aureus*, *E. coli*, and *B. cereus* and was able to inhibit the growth of *E. faecalis* and *K. variicola.* The paint containing CUR did not fulfill the requirements of JIS Z 2801 to be considered antibacterial; however, it was able to reduce the growth of *S. aureus, B. cereus*, and *E. faecalis* by almost 2-log.

The surfaces containing CLF or CUR presented low cytotoxicity after exposure of paint extracts to HaCaT and A549 cells. After incubation with the extracts, both surfaces presented good cellular viability, evaluated by WST-1 and NRU assays, as well as low interference with membrane integrity, evaluated with LDH assay. The test of direct contact performed with HaCaT cells also revealed high cellular viability and low LDH release after direct contact between the cells and the paints.

It is possible to say that our aim of developing non-cytotoxic paints with antibacterial properties based on natural compounds was achieved. Although more tests should be performed in the future—namely, to evaluate the paints’ efficacy on long term—the antibacterial properties of the paints incorporating these natural substances were demonstrated.

The extensive safety evaluation of the developed paints brings an advantage in the process of commercializing and applying the paints in real scenarios outside of the laboratory. Moreover, performing the necessary adaptations, it may even be possible to translate this antimicrobial technology and apply it on more products.

In conclusion, this study demonstrates the high potential of natural products to be applied on surfaces, giving them antimicrobial properties without compromising safety.

## Figures and Tables

**Table 1:** Antibacterial activity (R) values obtained for each bacterial specie after contact with CLF or CUR paint samples.

	*S. aureus*	*E. coli*	*B. cereus*	*E. faecalis*	*K. variicola*
CLF 3.2 g/L	2.9	2.0	3.2	1.6	0.6
CUR 0.4 g/L	1.5	1.0	1.9	1.6	0.7

## Data Availability

The data presented in this study are available on request from the corresponding author.
